# Variable versus fixed-rate infusion of phenylephrine during cesarean delivery: a randomized controlled trial

**DOI:** 10.1186/s12871-019-0879-3

**Published:** 2019-11-03

**Authors:** Ahmed Hasanin, Sara Habib, Yaser Abdelwahab, Mohamed Elsayad, Maha Mostafa, Marwa Zayed, Mohamed Maher Kamel, Kareem Hussein, Sherin Refaat, Ahmed Y. Fouda, Ahmed A. Wali, Khaled A. Elshafaei, Doaa Mahmoud, Sarah Amin

**Affiliations:** 1 Department of anesthesia and critical care medicine, Cairo university, Cairo, Egypt; 2 Department of anesthesia and critical care medicine, Ain Shams university, Cairo, Egypt; 3 Department of obstetrics and gynecology, Cairo university, Cairo, Egypt; 4 Department of anesthesia and critical care medicine, faculty of medicine, 01 elsarayah street, Elmanyal, Cairo, 11559 Egypt

**Keywords:** Hypotension, Cesarean delivery, Phenylephrine

## Abstract

**Background:**

Phenylephrine is the most commonly used vasopressor for prophylaxis against maternal hypotension during cesarean delivery; however, the best regimen for its administration is not well established. Although variable infusion protocols had been suggested for phenylephrine infusion, evidence-based evaluation of variable infusion regimens are lacking. The aim of this work is to compare variable infusion, fixed on-and-off infusion, and intermittent boluses of phenylephrine for prophylaxis against maternal hypotension during cesarean delivery.

**Methods:**

A randomized controlled study was conducted, including full-term pregnant women scheduled for elective cesarean delivery. Participants were divided into three groups which received phenylephrine by either intermittent boluses (1.5 mcg/Kg phenylephrine), fixed on-and-off infusion (with a dose of 0.75 mcg/Kg/min), or variable infusion (with a starting dose of 0.75 mcg/Kg/min). The three groups were compared with regard to frequency of: maternal hypotension (primary outcome), second episode hypotension, reactive hypertension, and bradycardia. Other outcomes included heart rate, systolic blood pressure, physician interventions, and neonatal outcomes.

**Results:**

Two-hundred and seventeen mothers were available for final analysis. The 2 infusion groups showed less incidence of maternal hypotension {26/70 (37%), 22/71 (31%), and (51/76 (67%)} and higher incidence of reactive hypertension compared to the intermittent boluses group without significant differences between the two former groups. The number of physician interventions was highest in the variable infusion group compared to the other two groups. The intermittent boluses group showed lower systolic blood pressure and higher heart rate compared to the two infusion groups; whilst the two later groups were comparable.

**Conclusion:**

Both phenylephrine infusion regimens equally prevented maternal hypotension during cesarean delivery compared to intermittent boluses regimen. Due to higher number of physician interventions in the variable infusion regimen, the current recommendations which favor this regimen over fixed infusion regimen might need re-evaluation.

## Introduction

Subarachnoid block is the preferred route of anesthesia during cesarean delivery. Maternal hypotension is a frequent and deleterious complication after subarachnoid block in this population. Without prophylactic vasopressors, the post-spinal hypotension affects nearly 60% of mothers undergoing cesarean delivery [1, 2]. Using vasopressors for prophylaxis against maternal hypotension have become fundamental in modern anesthetic practice [3, 4].

Phenylephrine (PE) is still the most commonly used vasopressor during cesarean delivery [3, 4]; however, the most appropriate protocol for PE administration is still unknown. PE is usually administered as single shot [5], fixed (on-off) rate infusion [6], or variable rate infusion [7]. The objective of all protocols is to achieve the least possible incidence of maternal hypotension, and avoiding reactive hypertension, with the least number of physician interventions. Continuous infusion regimens of PE are suggested to provide less incidence of hypotension compared to single bolus; however, stable hemodynamic profile requires a balance between preventing hypotension and avoiding unnecessary hypertension. Thus, reaching a definitive ideal regimen should be based upon this balance. To the best of our knowledge, no studies had previously compared variable rate and fixed (on-off) rate protocols for PE infusion during cesarean delivery. In this study, we compared variable infusion rate (at a starting rate of 0.75 mcg/Kg/min), fixed (on-off) rate (0.75 mcg/Kg/min), and intermittent boluses regimen (1.5 mcg/Kg) for prophylaxis against maternal hypotension during cesarean delivery.

## Methods

A randomized, controlled, trial was conducted in Cairo University hospital after approval of institutional research ethics committee approval (N-71-2107) on 16 September 2017. The study was registered at www.clinicaltrials.gov protocol registry system before enrolment of the first participant (Date of registration: 4 October 2017, clinical trial identifier: NCT03302039, principal investigator: Ahmed Hasanin). Written informed consent was obtained from all participants prior to recruitment in the study. The study was conducted from October 2017 to October 2018. An online random number software was run by a statistician to generate computer-generated sequence of codes (in a ratio of 1:1:1) which were placed into sealed, opaque envelopes. Each envelope included the instructions of preparing the drug, calculating the starting infusion rate, and the management protocol. The envelope was opened by a research assistant who was responsible for preparation of the drugs. Included participants were: full term, singleton, ASA physical status I or II, pregnant women scheduled for elective cesarean delivery, aged between 18 and 40 years. We excluded patients with cardiac morbidities, hypertensive disorders of pregnancy, peripartum bleeding, baseline heart rate < 60 bpm, and body mass index > 35 kg/m^2^. Upon arrival to the operating room, an 18G intravenous catheter connected to a three-way stopcock was inserted, and monitors were applied (electrocardiography – pulse oximetry – non-invasive blood pressure monitor). Baseline systolic blood pressure (SBP) was calculated as the average of three consecutive measurements at 2-min intervals with a difference < 10%. Patients were pre-medicated with metoclopramide (10 mg intravenous) and ranitidine (50 mg intravenous). Subarachnoid block was performed in the sitting position with 500 mL rapid co-load of lactated Ringer’s solution. Ten milligrams hyperbaric bupivacaine (2 mL) in addition to 20 mcg fentanyl were injected at L3-L4 or L4-L5 interspace using 25G spinal needle. Maternal blood pressure and heart rate were obtained at the baseline; then at 2-min interval till delivery of the fetus; then at 5-min intervals till the end of the operation. Block success was confirmed using pinprick. The surgeon was allowed to start the surgery when the block level was at T4. Patients with failed block (defined as sensory level below T4) were excluded from the study. Lactated Ringer’s solution was infused until a maximum of 1.5 l. Participants were placed in supine position with left uterine displacement. After spinal block, patients received PE according to the group allocation: Intermittent boluses group (*n* = 75): received 1.5 mcg/Kg PE bolus after spinal block, then received additional vasopressor boluses whenever SBP decreased by 20% or more from the baseline reading. Fixed infusion group (n = 75): received PE infusion with a starting dose of 0.75 mcg/Kg/min. If the blood pressure decreased by 20% or more, a vasopressor bolus was given without changing the rate of PE infusion. The infusion stopped when SBP increased by 20% or more from the baseline reading; when the SBP return to ±20% of the baseline reading, PE infusion was resumed. Variable infusion group (n = 75): received PE infusion at a starting dose of 0.75 mcg/Kg/min. The infusion was titrated up and down according to blood pressure as follows: 1- If the blood pressure decreased by 20% or more, a vasopressor bolus was given in addition to increasing PE infusion rate by 20%. 2- The infusion was stopped if blood pressure increased by 20% or more from the baseline; and was resumed in 50% of the baseline rate when SBP returned within ±20% of the baseline reading. For the infusion groups, the study drug was prepared in a 50-mL syringe (50 mcg/mL) and was administrated in the same line with running fluids. Vasopressor infusion was initiated just before injection of the local anesthetic in cerebrospinal fluid. Vasopressor infusion was stopped 5 min after delivery of the fetus. An oxytocin bolus (0.5 IU) was slowly administered after fetal delivery followed by an infusion of 2.5 IU/hour. Maternal hypotension (defined as SBP ≤ 80% of the baseline reading during the period from intrathecal injection to delivery of the fetus) was treated by PE bolus of 50 mcg. Severe maternal hypotension (defined as decreased SBP ≤ 60% of the baseline reading) was managed by PE bolus of 100 mcg. Bradycardia (defined as heart rate less than 55 bpm) was managed by IV bolus of ephedrine 9 mg (if accompanied with hypotension). If bradycardia was accompanied with hypertension, it was managed by stoppage of PE infusion. Atropine sulphate bolus (0.5 mg) was given if bradycardia was severe and persistent despite stoppage of the infusion.

### Primary outcome

Incidence of maternal hypotension: percentage of mothers with SBP ≤ 80% of the baseline preoperative reading during the period from spinal block till fetal delivery.

### Secondary outcomes

- Incidence of severe maternal hypotension (SBP ≤ 60% of the baseline preoperative reading during the period from spinal block till fetal delivery)
- Incidence of second episode hypotension (SBP ≤ 80% of the baseline reading after a previously managed episode)
- Incidence of post-delivery hypotension (SBP ≤ 80% of the baseline reading after fetal delivery)
- Incidence of reactive hypertension (SBP ≥ 20% the baseline preoperative reading after PE administration)
- Hemodynamic variables: SBP and heart rate (baseline reading and subsequent 12 readings)
- Number of physician interventions: each of the following was considered intervention: vasopressor bolus plus increasing the vasopressor infusion rate = 1 intervention, atropine bolus = 1 intervention, cessation of the vasopressor infusion = 1 intervention, re-starting of the vasopressor infusion.
- Intraoperative requirements of PE, ephedrine, and atropine.
- Incidence of intraoperative nausea and vomiting
- Neonatal data: Apgar score at 1 and 5 min post-delivery

### Statistical analysis and sample size calculation

Our primary outcome is the frequency of post-spinal hypotension. According to previous reports [5] the frequency of maternal hypotension was 37% in mothers who received the same PE bolus. Using G-power Software (version 3.1.9.2), a sample size of 170 participants was calculated to detect a difference of 20% in the frequency of hypotension, with a study power of 80%, and alpha error of 0.05. However, to allow the comparisons between the control group and each treatment group, an adjusted *P* (Bonferroni correction) of 0.025 was considered significant for the primary outcome; hence, the minimum sample size increased to 202 mothers (67 patients per group). The randomized number of participants was increased to 76 mothers per group for dropout compensation. Data analysis was performed using Statistical package for social science (SPSS) software, version 15 for Microsoft Windows (SPSS Inc., Chicago, iL, USA). Categorical data were presented as percentage and were analyzed using chi-squared test. Continuous Data were evaluated for normality through inspection of the histogram and were presented as mean (standard deviation) (for normally distributed data), or median (quartiles) (for skewed data). Inter-group comparisons for continuous data with normal distribution were conducted using one-way analysis of variance (ANOVA) test with post-hoc Tukey modification; whilst, skewed data were analyzed using Kruskal-Wallis on ranks. For analysis of repeated measures, two-way ANOVA test was run to evaluate groups (between-groups factor) and time (repeated measures). Bonferroni test was used for Post-hoc pairwise comparison. A *p* value of ≤0.05 was considered statistically significant.

## Results

**Fig. 1:**
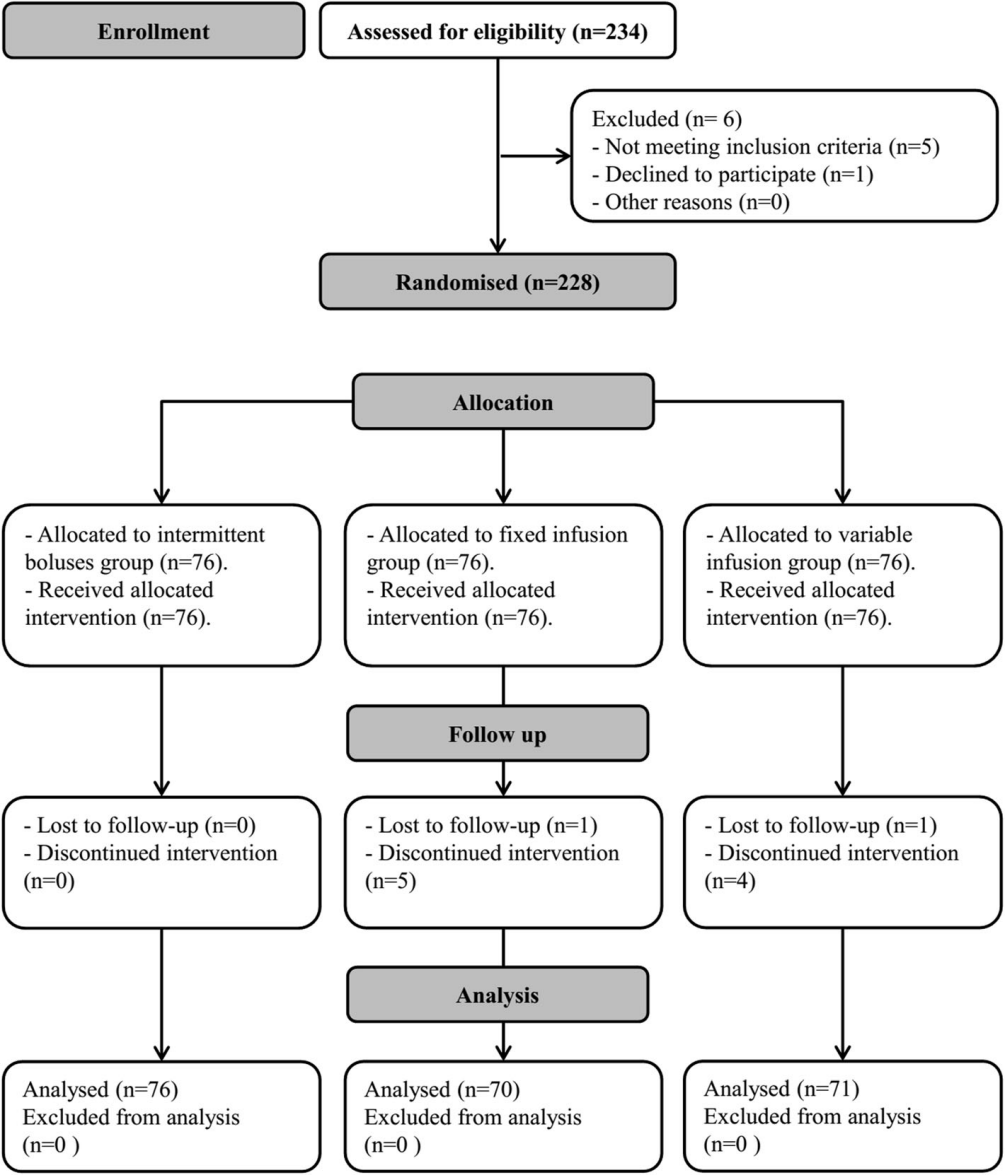
Flow chart for patient recruitment

**Fig. 2:**
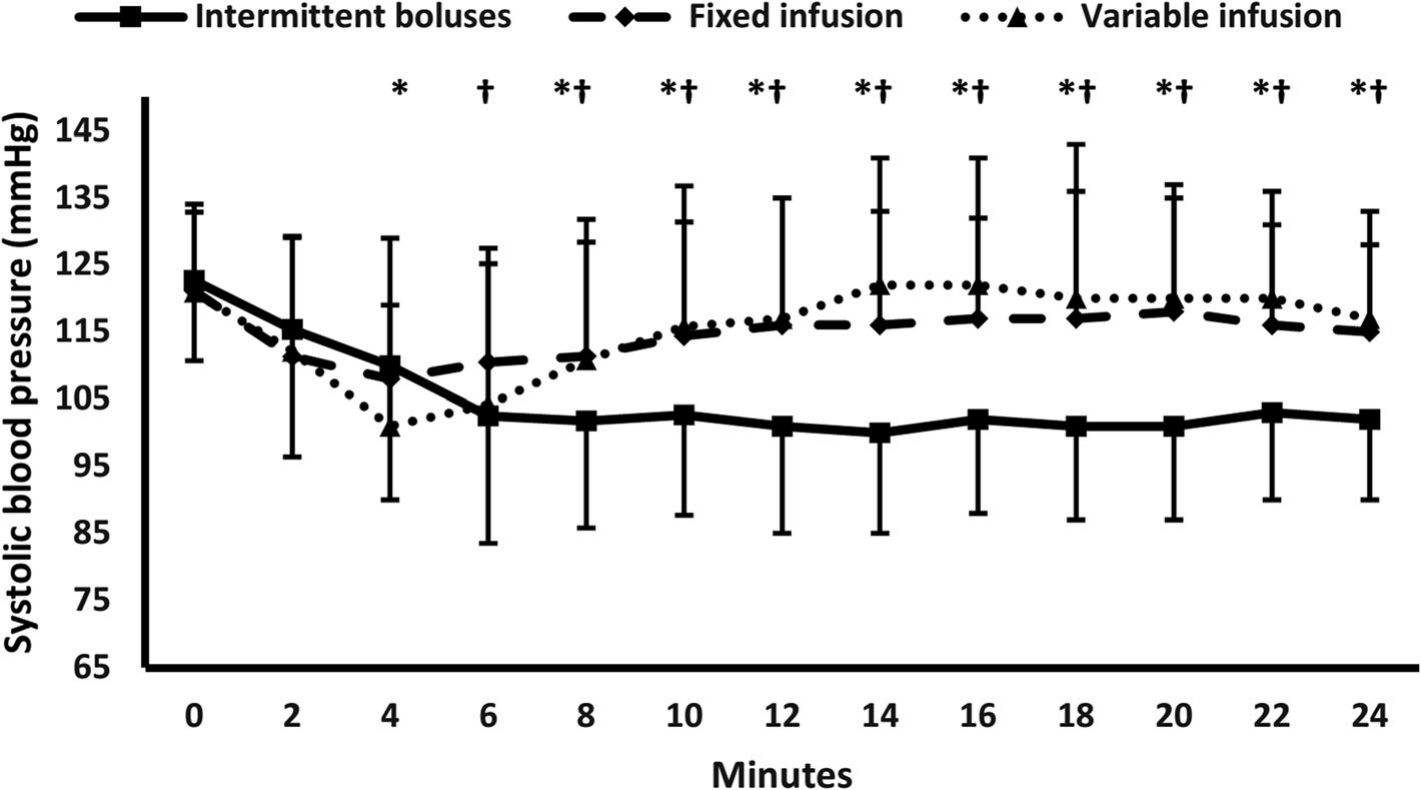
Systolic blood pressure. * denotes significance between single bolus group and variable infusion group. † denotes significance between intermittent boluses group and fixed infusion group

**Fig. 3:**
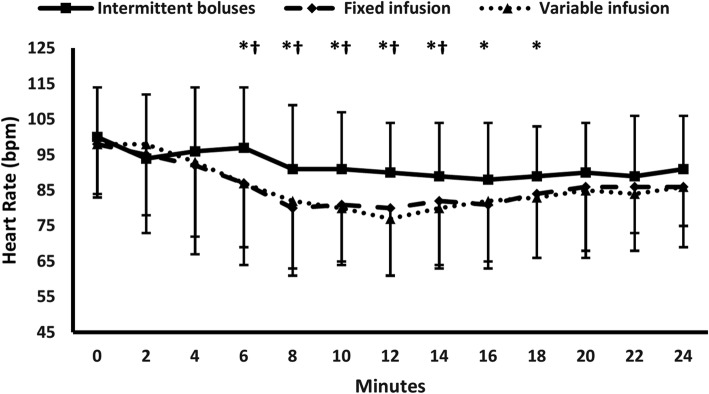
Heart rate * denotes significance between single bolus group and fixed infusion group. † denotes significance between intermittent boluses group and variable infusion group

Two-hundred and thirty-four mothers were screened for eligibility. Six mothers were excluded, 228 patients were randomized to receive one of the three interventions, 11 participants did not complete the intervention (failed spinal anesthesia – equipment failure), and 217 participants were available for final analysis (Fig. 1). Demographic data and baseline characteristics were comparable among the three groups (Table 1). Each of the fixed-infusion group and the variable-infusion group showed lower frequency of maternal hyptension and higher frequency of bradycardia compared to the intermittent boluses group without any significant difference between the two former groups {26/70 (37%), 22/71 (31%), and (51/76 (67%)}, and {12/70 (17.2%), 17/71 (23.9%), and (6/76 (7.9%)} (Table 2). Both infusion groups had also higher SBP, and lower heart rate compared to the intermittent boluses group at all readings without any significant difference between the two former groups (Figs. 2,3). The two infusion groups showed decreased SBP compared to their baseline readings in the first 10 min after subarachnoid block. The intermittent boluses group showed decreased SBP compared to the baseline values in all readings (Fig. 2). The 3 study groups had a decreased heart rate compared to their baseline values in most readings (Fig. 3). The number of mothers who had a second hypotensive episode after the initial vasopressor bolus was lower in the two infusion regimen groups compared to the intermittent boluses group without significant difference between the two infusion groups {(9/70(13%), 6/71(7%), and 25/76(33%), *P* < 0.001} (Table 2). The frequency of reactive hypertension was higher in the variable-infusion group compared to the intermittent boluses group; however, each of variable-infusion group and fixed-infusion group were comparable in reactive hypertension (Table 2).

**Table 1:** Demographic data, operative data, and baseline characteristics. Data are presented as mean (standard deviation), median (quartiles), and frequency (%)

	Intermittent boluses group (*n* = 76)	Fixed infusion group (*n* = 70)	Variable infusion group (*n* = 71)
Age (years)	28 (4)	27 (5)	27 (4)
Weight (Kg)	76 (70,82)	75 (70,80)	74 (69,84)
Time from SAB to delivery of the fetus (minutes)	22 (18,26)	20 (17,25)	22 (18,26)
Block level	T4 (T4,T4)	T4 (T4,T4)	T4 (T4,T4)
Baseline vital signs			
– SBP (mmHg)	123 (12)	121 (13)	121(12)
– Heart rate (bpm)	100 (14)	98 (15)	98 (14)
Mothers who received ergot alkaloids	3 (3.9%)	2 (2.9%)	2 (2.8%)

*SAB* subarachnoid block, *SBP* systolic blood pressure

**Table 2:** Maternal outcomes Data are presented as frequency (%), and median (quartiles), and mean (standard devition)

	Intermittent boluses group (*n* = 76)	Fixed infusion group (*n* = 70)	Variable infusion group (*n* = 71)	P1	P2	P3
Maternal hypotension	51(67%)	26(37%)^a^	22(31%)^a^	0.001	0.001	0.48
Severe maternal hypotension	12(16%)	10(14%)	12(17%)	0.82	1	0.82
Post-delivery hypotension	5(7%)	6(9%)	4(6%)	0.76	1	0.53
Bradycardia	6(7.9%)	12(17.2%)^a^	17(23.9%)+a	0.15	0.03	0.44
Hypertension	0(0%)	4(6%)	8(11%)^a^	0.05	0.002	0.34
Nausea	5(7%)	4(6%)	4(6%)	1	1	1
Vomiting	3(3.9%)	1(1.4%)	1(1.4%)	0.6	0.62	1
Hypotensive episodes	1(0,2)	0(0,1)^a^	0(0,1) ^a^	0.001	0.001	0.36
Mothers with 2nd hypotensive episode	25(33%)	9(13%)^a^	6(9%)^a^	0.008	0.008	0.62
PE consumption (mcg)	150(100,208)	1750(1400,2200)^a^	1510(1200,2100)^a, b^	0.001	0.001	0.014
No. of physician interventions per mother	1.42(1.44)	1.1(1.44)	1.89(1.86) ^b^	0.081	0.21	0.008
Physician interventions						
– Zero	24(32%)	34(49%)^a^	22(31%)^b^	0.043	0.96	0.04
– > 1	27(36%)	20(29%) 11(16%)	34(48%) ^b^	0.38 1	0.14	0.03
– > 2	13(17%)		24(34%) ^ab^		0.02	0.02

*PE* phenylephrine, *ep*. episodes, ^a^ denotes statistical significance compared to single bolus group, ^b^ denotes statistical significance compared to fixed infusion group. P1: P value between intermittent bolus group and fixed infusion group, P2: *P* value between intermittent bolus group and variable infusion group, P3: *P* value between fixed infusion group and variable infusion group

The median (quartiles) number of physician interventions was higher in the variable infusion group compared to the other two groups {1(0,3), 1(0,2), and 1(0,2)} (Table 2). The frequency of zero intervention was highest the fixed-infusion group (49%) than the intermittent boluses group (32%) and the variable-infusion group (31%) (*P* < 0.001} (Table 2). The frequency of mothers who received more than two interventions was also lower in the two former groups (Table 2). The two infusion groups showed higher PE consumption compared to the intermittent boluses group. Variable infusion group showed lower PE consumption compared to fixed infusion group {1510(1200,2100) mcg, 1750(1400,2200) mcg, *P* = 0.014} (Table 2). All other maternal and neonatal outcomes were comparable between the three study groups (Tables 2,3).

**Table 3:** Neonatal outcomes. Data are presented as median (quartiles), and frequency (%)

	Intermittent boluses group (*n* = 76)	Fixed infusion group (*n* = 70)	Variable infusion group (*n* = 71)
Umbilical artery *p*H	7.31(7.28,7.34)	7.31(7.28,7.34)	7.30(7.25,7.33)
Umbilical artery Pco_2_ (mmHg)	49(44,55)	46(42,52)	49(44,53)
Umbilical artery Po_2_ (mmHg)	24(17,30)	25(17,30)	24(19,28)
Apgar score at 1 min	9(8,9)	8(7,9)	9(7,9)
Apgar score < 7 at 1 min	4(5%)	5(7%)	5(7%)
Apgar score at 10 min	10(10,10)	10(9,10)	10(10,10)

## Discussion

We reported that both PE infusion protocols maintained maternal blood pressure better than intermittent boluses. Neither of the two infusion regimens (variable infusion regimen and fixed on-and-off regimen) was superior to the other in terms of hemodynamic outcomes (incidence each of hypotension, reactive hypertension, and bradycardia); however, the variable infusion regimen demonstrated more physician interventions. Vasopressor management during cesarean delivery aims to maintain a balance between prevention of hypotension and avoidance of reactive hypertension. Another aim is to be feasible and simple for the attending physician without the need for excessive un-necessary interventions. Many authors had used fixed on-and-off vasopressor infusion regimens effectively during cesarean delivery [6, 8–10]. Ngan Kee et al. demonstrated good results with 100 mcg/min in on-and-off infusion rate [8, 9]. Allen et al. had compared different doses for fixed PE infusion (25 mcg/min, 50 mcg/min, 75 mcg/min, and 100 mcg/min) and concluded that a dose of 25–50 mcg/min is a reasonable dose [6]. Variable infusion regimen of PE during cesarean delivery had been introduced by Siddik-Sayyid and colleagues [7] in a randomized controlled study. In their study, Siddik-Sayyid at al showed lower incidence of both PSH, and reactive hypertension with the variable infusion regimen, compared to the control group. However, Siddik-Sayyid et al. compared their new protocol to saline placebo with intermittent PE boluses. We suggest that proper evaluation of variable infusion PE regimen should be through its comparison to fixed on-and-off regimens which are already settled in obstetric anesthesia [11]. Our study is the first to compare both regimens (variable and fixed infusions) in addition to the presence of a control group which received intermittent PE boluses. We used the same protocol which was settled by Siddik-Sayyid and co-workers including the same starting dose. We selected a weight-based starting rate for PE in our patients. Selection of weight-based protocol versus fixed-dose protocol of vasopressors during cesarean delivery is another debatable issue. However, in a randomized controlled trial, weight-based vasopressor infusion protocol showed less hypotension compared to fixed-dose regimen [12]. The main management for a hypotensive episode is administration of a vasopressor bolus. Increasing the rate of the background vasopressor infusion was hypothesized to prevent subsequent hypotensive episodes. However, in our patients, the frequency of second hypotensive episode was comparable between both variable and fixed rates. Our findings provide an important piece of information that would impact future practice and research. As variable-infusion regimens were suggested to provide better maternal hemodynamic stability, the latest consensus had recommended to use variable-infusion rate in routine practice [3]. Our results suggest that using fixed on-and-off PE infusion would provide the same cardiovascular profile as variable infusion and would avoid much unnecessary physician interventions, and calculations. Our study has the advantage of being a randomized controlled study. Another advantage is the presence of a control group which received intermittent PE boluses. Our study had some limitations: 1- It is a single center study. 2- All our patients were scheduled for elective and not emergency cesarean delivery. 3- We did not include patients with cardiac morbidities. In conclusion, both infusion PE regimens equally prevented maternal hypotension during cesarean delivery compared to intermittent boluses regimen. Due to higher number of physician interventions in the variable infusion regimen, the current recommendations which favor this regimen over fixed infusion regimen might need re-evaluation.

## Abbreviations

ANOVA: Analysis of variance
ASA: American Society of Anesthesiologists
PE: Phenylephrine
SBP: Systolic blood pressure
SPSS: Statistical package for social science
